# Decision aid for patients considering total knee arthroplasty with preference report for surgeons: a pilot randomized controlled trial

**DOI:** 10.1186/1471-2474-15-54

**Published:** 2014-02-24

**Authors:** Dawn Stacey, Gillian Hawker, Geoffrey Dervin, Peter Tugwell, Laura Boland, Marie-Pascale Pomey, Annette M O’Connor, Monica Taljaard

**Affiliations:** 1 University of Ottawa, 451 Smyth Road, Ottawa, ON K1H 8M5, Canada; 2 Ottawa Hospital Research Institute, 501 Smyth Road, Ottawa, ON K1H 8L6, Canada; 3 University of Toronto and Women’s College Hospital, 76 Grenville Street, Toronto, ON M5S 1B2, Canada; 4 University of Montréal, 7101 Parc Avenue, Montreal, QC H3N 1X7, Canada; 5 Patient Decision Aid Research Group, Ottawa Hospital Research Institute, 501 Smyth Road, Ottawa, ON K1H 8L6, Canada

**Keywords:** Patient decision aids, Patient preferences, Osteoarthritis, Joint arthroplasty, Wait times, Decision quality

## Abstract

**Background:**

To evaluate feasibility and potential effectiveness of a patient decision aid (PtDA) for patients and a preference report for surgeons to reduce wait times and improve decision quality in patients with osteoarthritis considering total knee replacement.

**Methods:**

A prospective two-arm pilot randomized controlled trial. Patients with osteoarthritis were eligible if they understood English and were referred for surgical consultation about an initial total knee arthroplasty at a Canadian orthopaedic joint assessment clinic. Patients were randomized to the PtDA intervention or usual education. The intervention was an osteoarthritis PtDA for patients and a one-page preference report summarizing patients’ clinical and decisional data for their surgeon. The main feasibility outcomes were rates of recruitment and questionnaire completion; the preliminary effectiveness outcomes were wait times and decision quality.

**Results:**

Of 180 patients eligible for surgical consultation, 142 (79%) were recruited and randomized to the PtDA intervention (n = 71) or usual education (n = 71). Data collection yielded a 93% questionnaire completion rate with less than 1% missing items. After one year, 13% of patients remained on the surgical wait list. The median time from referral to being off the wait list (censored using survival analysis techniques) was 33.4 weeks for the PtDA group (n = 69, 95% CI: 26.0, 41.4) and 33.0 weeks for usual education (n = 71, 95% CI: 26.1, 39.9). Patients exposed to the PtDA had higher decision quality based on knowledge (71% versus 47%; p < 0.0001) and quality decision being an informed choice that is consistent with their values for option outcomes (56.4% versus 25.0%; p < 0.001).

**Conclusions:**

Recruitment of patients with osteoarthritis considering surgery and data collection were feasible. As some patients remained on the surgical waiting list after one year, follow-up should be extended to two years. Patients exposed to the PtDA achieved higher decision quality compared to those receiving usual education but there was no difference in wait for surgery.

**Trials registration:**

ClinicalTrials.Gov NCT00743951

## Background

Osteoarthritis is a common, disabling, and costly disease. Optimal medical management of osteoarthritis includes both non-pharmacologic and pharmacologic interventions [1]. For advanced disease, if medical therapy has failed, total joint arthroplasty (TJA) of the hip and knee is cost-effective [2-4]. However, referral for TJA is increasing due to an aging population. Wait times for surgery – both time to surgical consultation and to surgery once a decision is made to proceed - are unacceptably long in Canada [5-7]. Improving access while decreasing wait times for TJA is a priority for enhancing efficiency of care and patient satisfaction.

Previous efforts to reduce wait times for TJA have focused on the waiting period to undergo surgery, after the decision to proceed with a TJA is made with the surgeon. Such strategies include centralized waitlist management, triage, establishing maximum wait time benchmarks, increasing surgical capacity, and optimizing care pathways [5,8-11]. An important additional approach is to assess the patient’s informed preferences, prior to surgical consultation. In doing this, unnecessary referrals for surgical consultation may be avoided [12]. For instance, surgical candidates who never intend to have surgery would be identified prior to referral, thus decreasing the wait list [13-15]. Given that patient preferences for TJA are associated with their perceptions of the risks and benefits of the procedure, discussing risks and benefits when eliciting patient preferences can also ensure that those wanting surgery can access the proper pathways [15-20].

Unwillingness to consider TJA as a treatment option has been linked to misperceptions about the indications for, and risks and benefits of, TJA [15,16,21]. Patient preferences and perceptions about treatment options may be addressed using a patient decision aid (PtDA). PtDAs help patients become involved in health decisions by making the decision explicit, providing information about the options and their risks and benefits, and clarifying patients’ values. PtDAs are intended to be used in conjunction with regular consultation. A Cochrane review of 115 randomized controlled trials (RCT) showed that PtDAs increase patient participation in decision making, improve knowledge and realistic perceptions of benefits and harms, reduce decisional conflict, and improve the match between the chosen option and informed patients’ values [22]. PtDAs can have a substantive effect on over- and under-use of elective surgeries. In regions with high rates of surgical procedures (e.g., hysterectomy, discectomy, prostatectomy, coronary bypass surgery), PtDAs reduced preferences for surgical procedures by 20% without affecting health outcomes or patient satisfaction [22]. In regions where surgical rates were very low (e.g., prostatectomy in UK with shortage of urologists), the surgery rates increased. As such, PtDAs may have a role in ensuring that wait list reforms address under-use of surgical procedures that informed patients need and want, while preventing the over-use of procedures that informed patients do not value.

Two recent studies evaluated a PtDA focused on osteoarthritis with surgery and non-surgery options [23,24]. This PtDA is a booklet and DVD with high quality ratings based on the International Patient Decision Aid Standards [25]. Findings in one randomized controlled trial (RCT) revealed that compared to controls (n = 62) patients who used this PtDA (n = 61) felt more informed and confident in what to ask their doctor; the surgeons reported greater satisfaction and efficiency with the consultation and indicated that patients exposed to the PtDA asked more appropriate questions [23]. The other trial showed lower decisional conflict in those exposed to the PtDA (n = 70) compared to the PtDA with an adaptive conjoint analysis (n = 69) or educational booklet (n = 69) [24].

The objectives of this pilot RCT were to evaluate feasibility and to provide preliminary data on the effectiveness of the PtDA with a preference report for surgeons on wait times and decision quality in patients with osteoarthritis considering total knee replacement.

## Methods

### Design

A two-arm prospective RCT was conducted. Study approval was received from The Ottawa Hospital Research Ethics Board (# 2006724-01H) and the trial was registered (ClinicalTrials.gov, NCT00743951).

### Setting

Patients with osteoarthritis of the knee were recruited from an orthopaedic intake clinic, within a Canadian tertiary hospital. The sports medicine physician assessed surgical eligibility using the 7-item Western Wait List Hip Knee Priority Tool (HKPT) [8] mapped onto the three criteria for total knee arthroplasty according to the clinical practice guidelines (moderate to severe pain, moderate to severe functional limitations, and abnormal radiographic findings) [1]. Although, the priority tool was originally developed and validated as a transparent and fair approach for prioritizing patients on waitlists, it is used in this clinical setting as a standardized assessment tool applied to all patients. From April 2006 to March 2007, 47% of patients at this clinic were assessed to have milder osteoarthritis and were directed back to their referring physician with suggestions for conservative management [12]. The others were deemed eligible for surgical consideration for knee osteoarthritis.

### Participants

Eligible knee osteoarthritis patients were those with access to a television with a VCR or DVD player. Those with inflammatory arthritis, previous TJA, uncorrected hearing or visual impairment, or unable to read, or understand English, were excluded.

### Interventions

#### Experimental group

**Figure 1:**
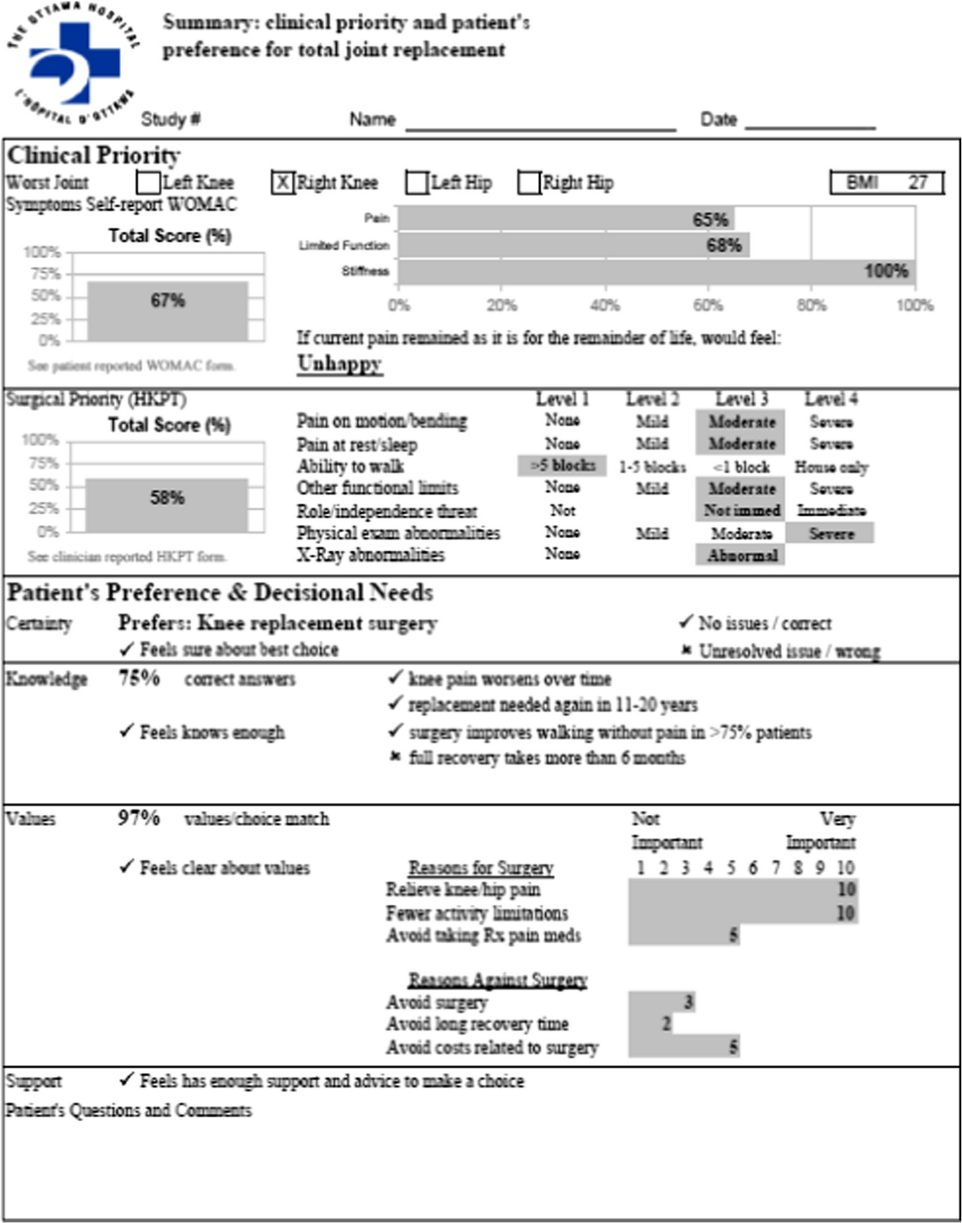
**Summary report for surgeons **[26]**.**

The PtDA, developed by the Informed Medical Decisions Foundation and distributed through Health Dialog, is entitled *Treatment Choices for Knee Osteoarthritis*. It consists of a 50-minute video and accompanying booklet that provides information on various treatment options for knee osteoarthritis, including lifestyle changes, non-drug treatments, pain medication, injections, complementary therapies, and surgery. A description of the options, probabilities of benefits and harms for each option, and video-clips of patient experiences allows patients to clarify their values associated with outcomes of options. According to the International Patient Decision Aid Standards, this PtDA meets most criteria for content (12 of 15), development process (8 of 9), and effectiveness (1 of 2). For more details on the IPDAS score card and the PtDA go to: http://decisionaid.ohri.ca/AZsumm.php?ID=1191. Patients received a questionnaire, formatted as user-friendly booklet, assessing their knowledge, values, preferred treatment choice, decisional conflict, and comments or questions. These results were combined with the patients’ clinical assessment findings to create a one-page preference report for the surgeon (see Figure 1) [26].

#### Control group

Patients in the usual education group received a standard information booklet prepared by the participating hospital for all patients undergoing joint replacement surgery. Information included preparation for surgery, recovery after surgery, and discharge plans. There was no information on benefits and harms of surgery or alterative options that could be used for decision making. Surgeons for patients in the control group received a half-page summary of patients’ clinical assessment findings only.

### Procedures

Eligible patients met with a research assistant who collected consent to participate in the study and baseline data. Baseline data included the Western Ontario McMaster Universities Osteoarthritis Index (WOMAC) measuring the patient’s perceptions of knee pain, stiffness and function, the HKPT completed by the assessing physician, and demographic information. This data was used to populate the surgeon’s clinical summary report (upper half of Figure 1).

After baseline data collection, patients were allocated to the PtDA intervention or usual education. The allocation schedule was computer-generated centrally by a statistician using a permuted block design with randomly varying block lengths of 4, 6, or 8. Allocations were concealed in numbered opaque sealed envelopes until after signed consent was obtained. Once allocated, patients were instructed to review their respective information (i.e., PtDA plus usual education booklet or usual education booklet only) at home and complete the accompanying questionnaire. Patients were not informed of the intervention characteristics. Although the research assistant was not blinded to group allocation, study outcomes for effectiveness were objective and obtained from clinic data (e.g. date of surgery or waitlist status).

Within two weeks from recruitment, the research assistant telephoned participants to obtain their answers to the questionnaire. This information was added to the surgeon’s clinical summary report to create the surgeon preference report (Figure 1). Participants were contacted by telephone one year after recruitment to determine whether they had seen the orthopaedic surgeon, and if so, whether they had chosen surgery or alternative non-surgical options. Dates for surgeon consultation and surgery were collected from the hospital health information system.

### Outcomes

The main feasibility outcomes were recruitment and questionnaire completion rates [27]. Feasibility targets were: a) >70% study enrollment of eligible patients; b) >90% of patients completing questionnaires at home prior to surgeon consultation; and c) <10% missing data. Preliminary effectiveness outcomes were wait times, decision quality, preparation for decision making, decisional conflict and patient feedback on the PtDA.

Preliminary effectiveness outcomes were assessed to inform a future larger scale RCT. Wait times were calculated based on the number of days from screening to definitive choices (i.e., date of surgery or date of decision to decline the surgery either explicitly stated or based on date appointment was cancelled without rebooking). Decision quality was defined as the extent to which patients’ decisions were informed and values congruent with their choice. Decision quality was deemed sufficient if a patient scored ≥66% on the knowledge test and if their predicted probability of surgery based on values corresponded with their actual choice. A score of 66% was chosen because the mean score for patients who had completed the knowledge test after viewing the PtDA was 68% [28] and it is consistent with knowledge scores in trials of PtDAs [22].

### Outcome measurement instruments

#### Hip-Knee Osteoarthritis Decision Quality Instrument

Patients’ knowledge was assessed using four multiple choice questions (i.e., osteoarthritis progress over time, need for revision joint replacement, proportion of patients with reduced pain, and length of time for recovery) from the Hip-Knee Osteoarthritis Decision Quality Instrument [28]. Knowledge scores were previously shown to be reproducible and to discriminate between those exposed to PtDAs and controls [28]. Patients’ values were measured by asking patients to rate the personal importance of the benefits and harms of outcomes for 6 items (e.g. relief of pain) on a 10-point rating scale with 1 indicating low importance and 10 indicating high importance. In a previous study, those who valued pain relief and return to normal activities were more likely to choose surgery while those who valued surgery avoidance were less likely to choose surgery [28]. The validity of these items have previously been demonstrated: patients whose treatment choice was concordant with their values felt more confident and had less regret with their decision [28].

#### Decisional Conflict Scale

The SURE tool, a 4-item version of the Decisional Conflict Scale, was used to assess patients’ perception of feeling sure, informed, supported, and clear about what mattered most [29]. In patients considering treatment options, this tool was previously shown to have adequate internal consistency with Kuder-Richardson 20 coefficient of 0.7 and significant correlation between the Decisional Conflict Scale and SURE scores [1].

#### Preparation for Decision Making

Four of the 10 items on the Preparation for Decision Making Scale [30] were used to determine patients’ perceptions of the decision making process. Four items were chosen because of their relevance to surgical decisions and they discriminated between patients prepared for decision making with PtDAs and those who were not. The items include recognition that a decision needs to be made (discrimination value 2.12), knowledge that the best choice depends on what matters most to the patient (3.39), level of decision making involvement desired by the patient (2.61), and patient preparedness for discussion with the surgeon (3.08). This instrument reports good internal consistency (>0.91) and excellent item discrimination (range 2.12 – 3.80) [30].

### Data management & statistical analysis

All data were entered twice into Microsoft Excel, verified for accuracy, and analyzed using SAS v. 9.1. Feasibility outcomes were summarized using descriptive statistics. Dichotomous effectiveness outcomes were describing using proportions with 95% confidence intervals and differences between arms were tested for statistical significance using chi-squared tests. Differences in the between groups median wait list times were described using Kaplan-Meier survival curves with 95% confidence intervals and were assessed for statistical significance using the log-rank test. Patients were censored at the end of the study, at the time of death, or loss to follow-up.

The mean scores on the knowledge test (calculated as the percentage of correct answers) were compared between the two groups using the two-sample t-test. The match between the patients’ choice and their values for benefits/risks was calculated as a dichotomous measure. The predicted probability of surgery was calculated for each patient using a logistic regression equation derived from three items assessing the patient’s values. The equation was [1 + exp (–*S*)]^–1^ where S = -0.3384 + 0.3869 × *Value Q*6 – 0.6111 × *Value Q*7 + 0.1933 × *Value Q*8 (Q6 relieve pain; Q7 avoid surgery; Q8 return to usual activities). The predicted probabilities were rounded to 0 or 1 (0 = no surgery and 1 = surgery) to determine the predicted choice based on the patient’s values. Mean scores on the Decisional Conflict Scale and Preparation for Decision Making scale were compared using the two-sample t-test. All tests were carried out at the two-sided 5% level of significance. Feedback from patients about the PtDA was analyzed descriptively.

## Results

### Feasibility

**Figure 2:**
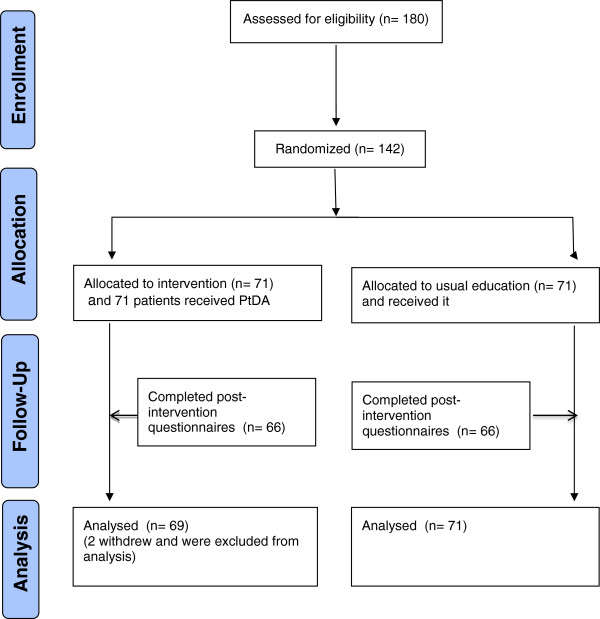
CONSORT trial flow diagram.

Of the180 patients deemed appropriate for surgical consultation between February 2007 and April 2008, 142 (78.9%) were eligible and consented to participate (see Figure 2). The most common reason for ineligibility was inadequate English. Of those eligible, 71 were randomized to the PtDA intervention group, 71 to usual education group. Sixty-six patients in both groups (93%) completed the questionnaires after exposure to the PtDA intervention and/or usual education materials. For those who completed the questionnaires, there was less than 1% missing responses for individual items. Two participants from the PtDA intervention group withdrew from the study and three participants from the usual education group were lost to follow-up. When supplemented with data from the hospital health information system, data on 140 (98.6%) patients was used for analysis of wait times.

Patient demographic characteristics indicate that the typical participant was 67 years of age, female, retired, and had completed secondary school (see Table 1). There were no statistically significant baseline differences between the groups based on demographic characteristics, WOMAC scores, or HKPT scores.

**Table 1:** Patient demographic characteristics

	**PtDA intervention**	**Usual education**
	**(n = 69)**	**(n = 68)**
Age (yrs), mean (SD)	67.1 (10.85)	67.3 (12.16)
HKPT* (total 80), mean (SD)	43.9 (12.4)	45.1 (17.5)
WOMAC* (total 96), mean (SD)	60 (0.17)	64 (0.18)
Men	19 (27.5%)	25 (35.2%)
Women	50 (72.5%)	46 (64.8%)
Education		
Less than secondary school	10 (14.5%)	12 (16.9%)
Secondary school/trades school	25 (36.2%)	30 (42.3%)
Post-secondary education	32 (36.4%)	24 (33.8%)
Missing	2 (2.9%)	5 (7.0%)
Retired	37 (53.6%)	42 (59.1%)

*higher scores indicate higher severity of osteoarthritis.

Overall, patients’ feedback about the PtDA was positive. Patients liked using the PtDA as a tool to communicate and share with others. Patient 40 said that the PtDA *“…helped my spouse to understand what I was going through*”. Comments indicated that patients liked the presentation and some said it helped them arrive at a decision. For instance, Patient 101 reported, *“…the information regarding total knee replacement was very good*” and Patient 97 indicated, “*This material was very helpful. It helped me to decide that of all the options available, knee replacement surgery is the best option in my case*.” Patients said the PtDA helped them learn the facts and have their questions answered. For example, Patient 117 said *“…the video clarified the points that I had questions on*” and Patient 96 highlighted, “*I have investigated this to death and found the tape very good.*” Finally, the PtDA helped patients understand that they had a role in decision making, which was supported by Patient 126’s comment, “*Excellent tool that really helped me understand the importance of my collaborative decision making with the health care team.”*

Ten patients provided negative feedback. Some wanted more information about “*…the wait time, the recovery time, and the consequences*” (Patient 98). Information that was described as confusing included when “*people were talking about personal stories*” (Patient 21), and “*pictures on the first page*” (Patient 24).

At the end of the 1-year follow-up period (May 2009), 55 of 69 patients in the PtDA group had undergone surgery (79.7%; 95% CI 70.2 to 89.2%), 5 chose non-surgical management, 8 were still on the waiting list for surgery, and 1 had died. Of 68 patients in the usual education group, 48 underwent surgery (70.6%; 95% CI 59.8 to 81.4%), 9 chose non-surgical management, 10 were still on the waiting list for surgery, and 1 had died. There was no statistically significant difference between groups in the proportions of patients undergoing surgery (difference between PtDA versus usual education 9.1%, 95% CI -5.3% to 23.5%, p = 0.2165).

### Preliminary effectiveness outcomes

**Figure 3:**
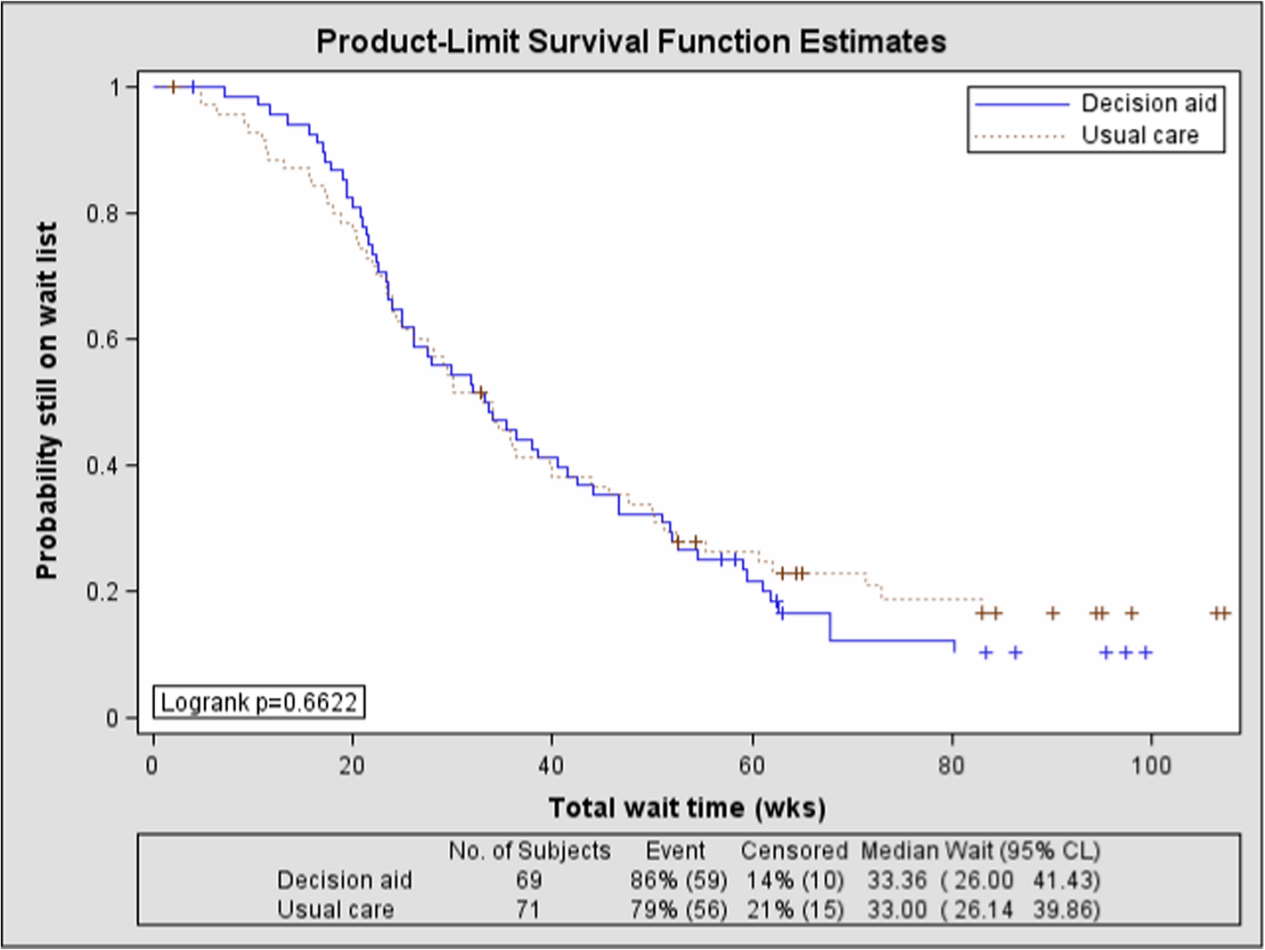
Wait times (screening to definitive decision) by group.

The median total wait times from the screening consultation to a definitive decision (e.g. underwent surgery or off wait list for non-surgical management) was 33.4 weeks for the PtDA group (n = 69, 95% CI: 26.0, 41.4) compared to 33.0 weeks for the usual education group (n = 71, 95% CI: 26.1, 39.9) (see Figure 3). There was no significant difference in the time on the wait list between groups (log-rank p = 0.6622).

Mean knowledge scores for patients who used the PtDA were 71.2% compared to 46.6% in the usual education group (p < 0.01) (see Table 2). Patients in the PtDA group were more likely to have a higher quality decision (informed choice that matched their values for outcomes of options) (31 of 55 (56.4%) versus 14 of 56 (25.0%); p < 0.001). After exposure to the intervention and prior to the surgeon consultation, 20 of 66 patients in the PtDA group (30.3%) were unsure of the best option (prefer surgery or prefer non-surgery) compared to 9 of 66 patients in the usual education group (13.6%) (p = 0.0208). There were no statistically significant differences between groups for any items on the Decisional Conflict Scale or Preparation for Decision Making scale.

**Table 2:** Preliminary effectiveness outcomes

	**PtDA**	**Usual education**
Knowledge, mean	n = 66	n = 66
	71.2%	46.6%
High quality decision, n (%)	n = 55	n = 56
	31 (56.4)	14 (25.0)
Uptake of chosen option at one year, n (%)	n = 69	n = 68
TJA surgery	55 (79.7)	48 (70.6)
No surgery	5 (7.2)	9 (13.2)
Waiting list	8 (11.6)	10 (14.7)
Died	1 (1.4)	1 (1.5)
Median total wait time from screening consultation to definitive decision	n = 69	n = 71
	33.4 weeks	33.0 weeks
SURE test scores* prior to surgeon consultation, n (%)	n = 65	n = 66
Feels SURE about best choice	47 (72.3)	53 (80.3)
Knows the benefits and harm of each option	60 (92.3)	44 (66.7)
Clear about which benefits and risks matter most	57 (87.7)	49 (74.2)
Has enough support and advice to make choice	50 (76.9)	51 (77.3)
TOTAL SURE score = 4	45 (69.2)	38 (57.6)
Preparation for decision making^†^, mean (SD)	n = 66	n = 64
Help recognize decision to be made	4.121 (1.209)	3.781 (1.253)
Help know decision depends on what matters most	4.477 (0.850)	4.141 (1.096)
Help think about how involved you want to be in decision	4.477 (0.812)	4.250 (1.054)
Prepare you to talk to your doctor about what matters most	4.364 (0.905)	4.234 (1.035)

*Those not responding YES are experiencing decisional conflict.

^†^scored on a 5-point scale from 1 (not at all) to 5 (a great deal).

## Discussion

### Feasibility and considerations for subsequent trial

Our study showed that it was feasible to recruit patients with knee osteoarthritis, administer the decision support interventions, and collect outcome measures. Patients in this study rated their experience using the PtDA favorably as indicated in both the preparation for decision making items and their qualitative feedback.

Surgical wait times were challenging to measure given the length of time patients wait for surgery in Canada. As reported earlier, 12% of patients in the PtDA group and 15% of patients in the usual education group were still waiting for surgery after one year. This suggests that using the reported Canadian averages to determine the duration of the study is not adequate for collecting follow-up measures. In fact, patients need to be followed for much longer to ensure accurate comparisons between groups.

Whether or not wait times is an appropriate primary outcome for a subsequent study must be considered. This study was originally designed to recruit patients prior to attending the surgical screening clinic but was changed during the ethics approval process to recruit patients in the clinic. Interestingly, almost half the patients assessed in this screening clinic were sent back to the referring physician because they were diagnosed with milder osteoarthritis and ineligible for this study [12]. Given that the PtDA included information on non-surgical management options for osteoarthritis and the significant improvement in knowledge among patients exposed to the PtDA, it may be more appropriate to recruit patients with various severity of osteoarthritis to the study instead of limiting recruitment to those eligible for surgical consultation.

### Preliminary effectiveness

Measuring decision quality as a composite measure was possible in this study. A quality decision, the ultimate goal of PtDAs, is ideally measured by using patient’s score on the knowledge test as an indicator of being informed, and measuring the concordance between the informed patient’s values for outcomes of options and the actual choice of surgery (or non-surgery) [25,31]. In this study, patients exposed to the PtDA intervention obtained significantly higher decision quality (56%) compared to those who received usual education (25%). These findings are consistent with a systematic review of 115 trials of PtDAs that showed a 51% higher improvement in informed values-based choices [22]. However, several trials included in the systematic review used ‘feeling clear about values’ rather than the actual measure of values-choice concordance.

Patients in the PtDA intervention group who had learned more about their options, benefits and harms reported more decisional conflict about their decision prior to consultation with the surgeon compared to the usual education group. Although we didn’t reassess decisional conflict after surgical consultation, there was no statistically significant difference between groups in the proportion of patients indicating they were unsure about the best option. The higher level of decisional conflict in patients exposed to PtDAs prior to surgical consultation is consistent with findings in a study of women with breast cancer considering surgical options [32]. Women considering options for breast cancer surgery had improved knowledge, better clarity of values and felt more supported, but were only sure of the best option after consultation with the surgeon. Patients need to discuss their values and preferences with the surgeon prior to feeling certain about the best treatment choice for them. Furthermore, PtDAs encouraged patients to use information on options including relevant benefits and harms when making a treatment choice [33], and as observed in this study achieving a higher quality choice. By helping patients achieve a higher quality decision and ensuring a positive decision making experience, they are more likely to avoid downstream decisional regret [34].

### Limitations

There are three main limitations to consider when interpreting the results of this study. In terms of designing a future trial, patients need to be followed for longer given that 13% were still waiting for surgery after one year. Using the results from the pilot study, the required sample size for a future definitive trial can be determined: 155 patients per group, followed over two years, would be required to detect a clinically important difference of 8 weeks in mean total wait times, using a two-sided t-test at the 5% level of significance with 80% power, assuming a common standard deviation of 25 weeks. To account for 10% loss to follow-up, we would need to enroll 173 patients per group. Also, decisional conflict and preferred option were only measured prior to seeing the surgeon. Given that a quarter of patients in the PtDA group felt unsure, a subsequent study should also measure decisional conflict after seeing the surgeon. Limitations related to the preliminary effectiveness outcomes are potential for self-report bias given that most outcome measures were patient reported. For the outcome of actual choice, self-report bias may have been mitigated because data was also extracted from the patients’ health record for verification of the data.

## Conclusions

This pilot RCT demonstrated a high patient recruitment rate and completed patient questionnaires with minimal missing data. Therefore, the feasibility targets were met, warranting future larger scale trial. Preliminary effectiveness outcomes demonstrated that the PtDA improved decision quality and knowledge. A subsequent trial should follow patients for longer than one year and repeat decisional conflict measures before and after surgical consultation. Findings were used to inform the design and redefine primary outcomes of a larger scale study.

## Abbreviations

TJA: Total joint arthroplasty of the hip and knee; RCT: Randomized controlled trial; PtDA: Patient decision aid; WOMAC: Western Ontario McMaster Universities Osteoarthritis Index; HKPT: Knee priority criteria tool.

## Competing interests

The authors (DS, GH, PT, IT, LB, MPP, AC, MT) declare that they have no competing interests. GFD is a paid consultant for Stryker Corporation advising on total and partial knee replacement.

## Authors’ contributions

All authors participated in the design of the study. DS was the primary investigator responsible for all parts of the study and drafted the manuscript. MT performed the statistical analysis. All authors read and approved the final manuscript.

## Pre-publication history

The pre-publication history for this paper can be accessed here:

http://www.biomedcentral.com/1471-2474/15/54/prepub
